# Selective Laser Melting of Hydroxyapatite: Perspectives for 3D Printing of Bioresorbable Ceramic Implants

**DOI:** 10.3390/ma14185425

**Published:** 2021-09-19

**Authors:** Natalia V. Bulina, Sergey G. Baev, Svetlana V. Makarova, Alexander M. Vorobyev, Alexander I. Titkov, Victor P. Bessmeltsev, Nikolay Z. Lyakhov

**Affiliations:** 1 Institute of Solid State Chemistry and Mechanochemistry, Siberian Branch of the Russian Academy of Sciences, Kutateladze Str. 18, 630128 Novosibirsk, Russia; makarova@solid.nsc.ru (S.V.M.); voralexmih@mail.ru (A.M.V.); a.titkov@solid.nsc.ru (A.I.T.); lyakhov@solid.nsc.ru (N.Z.L.); 2 Institute of Automation and Electrometry, Siberian Branch of Russian Academy of Sciences, Academician Koptyug Avenue 1, 630090 Novosibirsk, Russia; baev@iae.nsk.su (S.G.B.); bessvic@gmail.com (V.P.B.)

**Keywords:** bioceramics, hydroxyapatite, selective laser melting, sintering, 3D printing

## Abstract

Hydroxyapatite, being the major mineral component of tooth enamel and natural bones, is a good candidate for bone tissue engineering applications. One of the promising approaches for manufacturing of three-dimensional objects is selective laser sintering/melting which enables the creation of a dense structure directly during 3D printing by adding material layer-by-layer. The effect of laser irradiation with a wavelength of 10.6 μm on the behavior of mechanochemically synthesized hydroxyapatite under different treatment conditions was studied for the first time in this work. It was shown that, in contrast to laser treatment, the congruent melting is impossible under conditions of a relatively slow rate of heating in a furnace. Depending on the mode of laser treatment, hydroxyapatite can be sintered or melted, or partially decomposed into the more resorbable calcium phosphates. It was found that the congruent selective laser melting of hydroxyapatite can be achieved by treating the dense powder layer with a 0.2 mm laser spot at a power of 4 W and at a scanning speed of 700 mm/s. Melting was shown to be accompanied by the crystallization of a dense monolayer of oxyhydroxyapatite while preserving the initial apatite crystal lattice. The thickness of the melted layer, the presence of micron-sized pores, and the phase composition can be controlled by varying the scanning speed and laser power. This set of parameters permits the use of selective laser melting technology for the production of oxyhydroxyapatite biodegradable implants with acceptable properties by 3D printing.

## 1. Introduction

Restorative and reconstructive surgery is a critically important area of personalized medicine with the potential to design and manufacture individual bone implants that depend on the needs of each patient. Bone is a highly complex material that exhibits a strongly hierarchical structure on different length scales [1]. Moreover, the bone tissue of different parts of the body of a person has different porosity, and the composition of bone tissue can change with the age increased [2].

There are various methods for production of scaffolds with a porous internal structure, similar to that of the real bone tissue [3,4]. Shaping of the objects by the additive manufacturing techniques, also known as 3D printing technologies are very popular. All technologies of this type are based on a layer-by-layer building of an object (i.e. layer-by-layer printing). The most efficient way to print individual bone implants with specific shape through additive technologies is selective laser sintering (SLS) or selective laser melting (SLM) being one of the most developed and successfully applied methodologies for today [5,6,7]. This technology has a number of advantages, such as local concentration of energy, ultrafast heating and cooling, easy control, and the ability to create objects with designed complex shape and developed porous structure [5,6,8]. This approach also allows one to avoid intense shrinking of the printed object which takes place at high temperature post-treatment when using other 3D printing techniques.

Hydroxyapatite (HA), with the chemical formula Ca_10_(PO_4_)_6_(OH)_2_, is the main mineral component of tooth enamel and bone. Among various calcium phosphates, the synthetic apatite is one of the most widely used in medical applications [1,9]. In addition, HA is one of the promising materials for the fabrication of ceramic bioresorbable scaffolds to stimulate new bone ingrowth [10] and it is also actively used as additive to produce 3D printable composite materials with high biocompatibility [11,12,13]. In most studies, when printing bioresorbable scaffolds, HA is added to an organic polymer. Depending on the printing method, this is either a binder (three-dimensional printing (3DP)), UV-polymerizable resins (stereolitography (SL)), or thermoplastic polymers (fused deposition modeling (FDM)) [5,13]. In all these methods, the product, its unique geometry and structure are actually formed due to the polymer component, while the hydroxyapatite itself is just a filler of the polymer composite. Further, the organic polymer, in most cases with the exception of biodegradable polymers such as PLA and its derivatives, must be removed by heat treatment [10,12,14]. This complicates the manufacturing process, leading to geometric distortion and deformation of the unique structure of the final product caused by shrinkage, and contaminates the product with carbon-based polymer residues [14].

Some resent studies reported the possibility of printing of HA scaffolds in the absence of additives/fillers using SLS technology [15,16,17]. It has been shown that this technique allows apatite particles to be sintered without decomposition and, thus, to carry out layer-by-layer printing of a three-dimensional object. However, in these studies, the size of the laser spot used to treat the surface of the powder was 1–4 mm. Therefore, the resolution of the sintered parts was not sufficient. To fabricate scaffolds simulating the cancellous bone structure, a resolution of 0.1 mm is required [14]. In addition, the scanning speed of hundreds of millimeters per minute is very low and unacceptable when fabricating real objects using additive manufacturing technologies [14].

**Figure 1:**
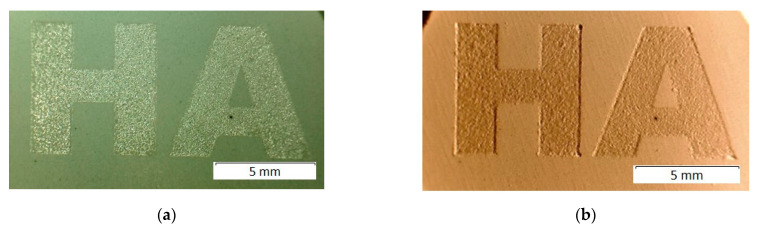
General view of the surface of the pellet partially treated with laser irradiation. Lighting from the top (**a**) and from the right side (**b**).

Earlier we showed [18] that congruent melting of HA occurred under the CO_2_ laser treatment with a power of 4 W for a spot size of 0.2 mm and a scanning speed of 640 mm/s and resulted in the formation of a glassy coating on the surface of the compacted pellet (Figure 1). It was found that due to the low bulk density of HA, a uniform layer could not be obtained by melting the bulk layer of the HA powder. Therefore, when dealing with the HA powder, the density of the treated layer should be increased by means of compacting or adding a small amount of a highly volatile liquid to the powder. To obtain a uniform layer of molten HA, the density of the powder should be not less than 1.9 g/cm^3^. 

It should be noted that, during sintering, linear shrinkage of the HA compact can reach 20% [19]. In this regard, thermal post-treatment of the HA products leads to significant shrinkage and deformation, which does not allow using an individual approach in the production of medical HA implants. When using selective laser melting, widely used for printing metal implants, shrinking occurs directly upon melting of the HA layer during printing the object, as seen in Figure 1b.

The aim of this work is to find the optimal parameters for laser treatment to obtain a dense monolayer of the recrystallized HA for further application in layered 3D printing of personal ceramic implants.

## 2. Material and Methods

The HA powder was synthesized by the dry mechanochemical method via mechanical treatment of the reaction mixture of СаНРО_4_ and just calcined CaO powders. The reagents were mixed in ratios according to the following Reaction:

6CaHPO_4_ + 4CaO → Ca_10_(PO_4_)_6_(OH)_2_⋅2H_2_O (1)

The mechanical treatment of mixture was carried out in the planetary ball mill, AGO-2, equipped with water-cooled steel vials. The reaction mixture was treated by steel balls weighing 200 g at a rotational speed of 1800 rpm. The duration of the synthesis was 30 min. The specific surface area of the as-synthesized powder was 18 m^2^/g.

Compacting the HA pellets with a diameter of 18 mm was carried out at a pressure value of 3 tons using a manual hydraulic press. The density of the pellets obtained was 1.9 g/cm^3^. Sintering of the pellets was carried out in a high-temperature electrical furnace PVK-1.6-5 at different temperatures during 10 min using a heating rate of 5 °C/min.

The HA pellet surface was irradiated with a CO_2_ laser operating at a wavelength of 10.6 μm, using the experimental unit developed by the Institute of Automation and Electrometry SB RAS (Novosibirsk, Russia). An optical-mechanical 2D scanner with Fθ lens was used to scan and focus the laser beam [20]. During laser treatment, the sample was placed on a Z-axis motion platform made from aluminum and moved in the vertical direction. The samples were irradiated by raster scanning of the surface with a focused laser beam in the program control mode. Scanning was performed at fixed values of the laser treatment parameters presented in Table 1. The focused laser spot for used laser has a non-uniform near-Gaussian intensity distribution with intensity level exp(-2). The laser spot size was determined experimentally by recording lines on a thermosensitive medium. During the experiments, a white glowing spot was observed on the surface of the exposed HA sample. The presence of a glassy coating after laser irradiation is an indirect visual indicator for achieving the exposure required to melt the sample surface (Figure 1a).

The laser exposure was assessed according to the Equation:

*E = P/(V⋅d)* (2)

where *E* is the exposure value, *P* is the laser power, *V* is the speed of laser spot moving, and *d* is the raster pitch. An estimation using this formula is allowed in raster-scanning mode with a raster pitch of significantly less than the laser spot size (i.e., scanning with high overlapping to average the effect of a Gaussian laser beam).

The morphology of the samples was analyzed using scanning electron microscopes Hitachi TM1000 and Hitachi 3400N. To obtain cross sectional images of the samples, the tablets were broken without further processing. X-ray diffraction (XRD) patterns of the samples obtained were recorded on a Bruker D8 Advance powder diffractometer with Bragg-Brentano geometry using Сu Kα radiation. X-ray phase analysis of the samples was carried out using PDF-4 database (ICDD, Release 2011). The unit cell parameters and crystallite size were refined by the Rietveld method using Topas 4.2 software (Bruker AXS, Karlsruhe, Germany). FTIR spectra were recorded on an Infralum−801 spectrometer using the KBr pellet method. Differential thermal analysis was carried out using a Netzsch STA 449 F1 Jupiter device equipped with a QMS 403 C Aeolos mass-spectrometer. The measurements were performed in a Pt–10wt.-%Rh crucible under an argon-oxygen mixture (80:20) at a heating rate of 10 °C/min.

## 3. Results and Discussion

### 3.1. Sintering in a High Temperature Furnace

**Figure 2:**
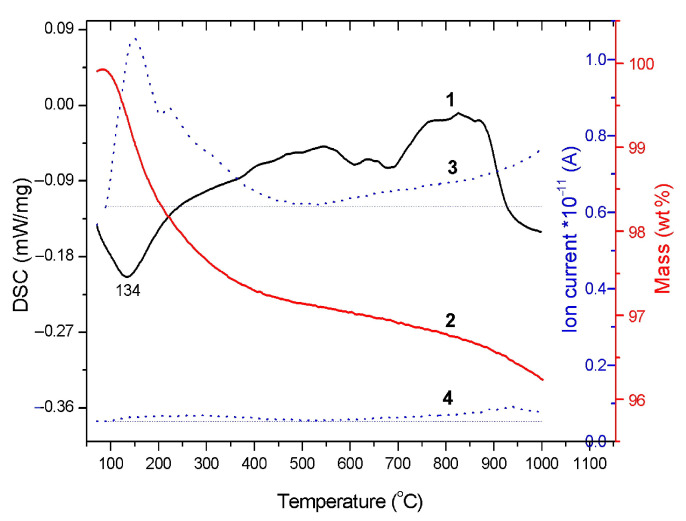
Thermal analysis of the as-synthesized HA powder: 1, differential scanning calorimetry; 2, weight loss; 3, evolved water; 4, evolved carbon dioxide.

The results of thermal analysis show (Figure 2) that during heating the HA powder in the temperature range 25–1000 °C, a weight loss of up to 4% occurs. The main loss is due to the removal of water. A small amount of water released during the HA synthesis by Reaction (1) is adsorbed on the particle surface filling the cracks and pores; in addition, it incorporates into the apatite lattice. During heating, the adsorbed water is removed in the temperature range from 25 to 200 °C, while the lattice water is released between 200 and 500 °C. Consequently, the endothermic effect at 134 °C observed on the differential scanning calorimetry (DSC) curve (Figure 2) corresponds to evaporation of the adsorbed water. Between 400 and 900 °C, structural ordering occurs, which is also accompanied by the release of a small amount of structural water. An endothermic effect on the DSC curve started from 880 °C corresponds to the dehydroxylation of HA [21] with the removal of water and the formation of oxyhydroxyapatite (OHA) according to the following Reaction:

Ca_10_(PO_4_)_6_(OH)_2_ → Ca_10_(PO_4_)_6_(OH)_2–2x_O_x_□_x_ + xH_2_O (3)

where □ is the hydrogen vacancy.

The crystal structure of OHA is similar to that of HA, therefore, HA dehydroxylation is not accompanied by any structural changes. However, due to the low stability of OHA, the reverse process, i.e., rehydroxylation, occurs upon its cooling in a water vapor atmosphere [21]. Therefore, after heating HA up to 1200 °C and cooling the sample in the presence of water molecules, its stoichiometric composition should be Ca_10_(PO_4_)_6_(OH)_2_.

In accordance with Reaction (3), the more water molecules release, the fewer hydroxyl groups remain in the apatite structure. At a limiting case of x = 1, the apatite structure has no hydroxyl groups and, in this case, an oxyapatite (OA) Ca_10_(PO_4_)_6_O is formed. An increase in the temperature above 1300 °C leads to the OA decomposition by the following Reaction [21]:

Ca_10_(PO_4_)_6_O → 2Ca_3_(PO_4_)_2_ + Сa_4_O(PO_4_)_2_ (4)

**Figure 3:**
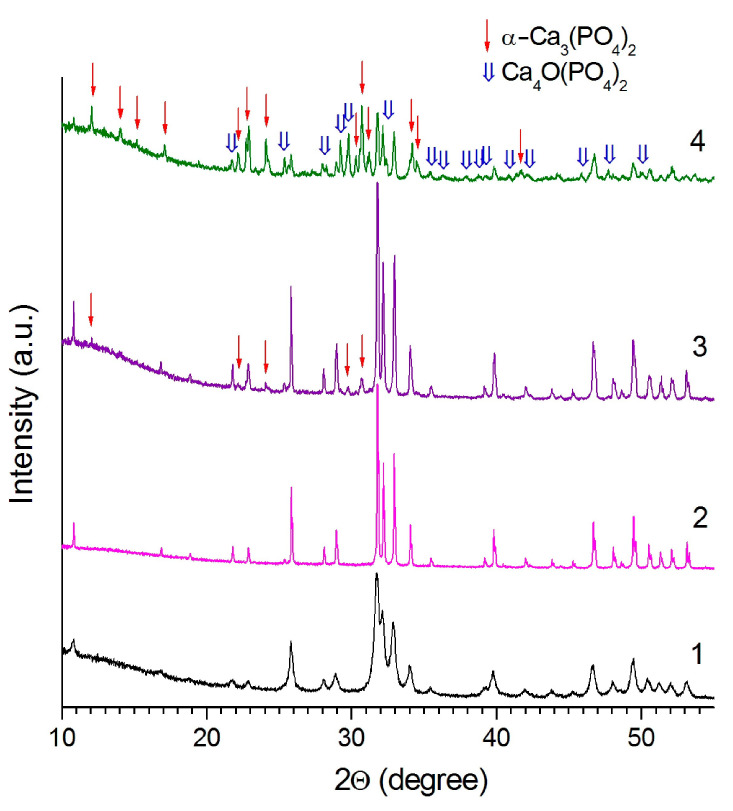
XRD patterns of the initial HA pellet (1) and the HA pellet sintered into the high-temperature furnace at temperatures: 2, 1300 °C; 3, 1400 °C; 4, 1500 °C.

According to the XRD data, the HA synthesized by the mechanochemical method retains its structure up to a temperature of 1300 °C (Figure 3), which is consistent with the thermal stability of HA synthesized by other methods [21]. Reflections of the impurity phase α-Ca_3_(PO_4_)_2_ appear in the sample, when the temperature reaches 1400 °C. At 1500 °C, the intensity of the α-Ca_3_(PO_4_)_2_ reflections increases, and a reflection of tetracalcium phosphate Ca_4_O(PO_4_)_2_ appears in the XRD pattern indicating the partial HA decomposition by Reaction (4). A similar behavior in a high-temperature furnace took place for HA prepared by other synthesis methods [21,22].

**Figure 4:**
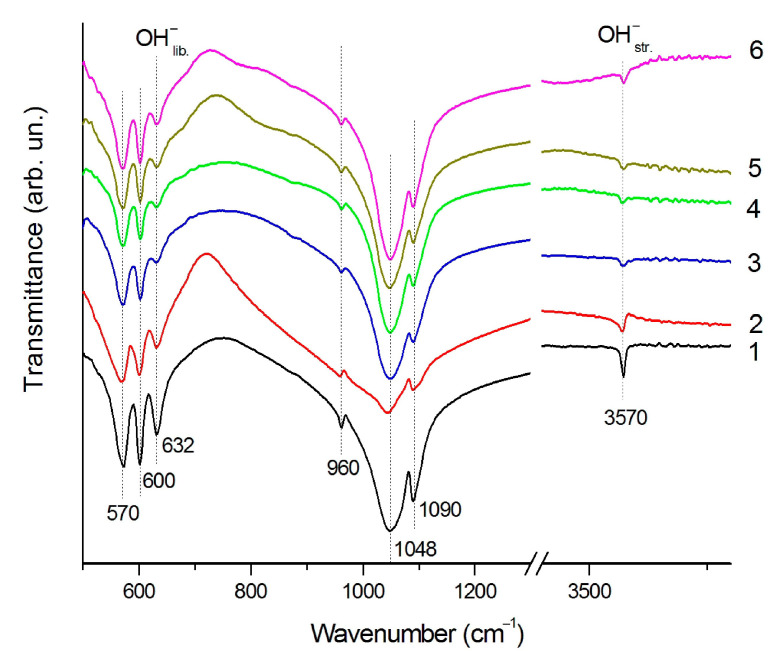
FTIR spectra of HA samples heated at 900 °C (**1**) and 1400 °C (**2**) into furnace and treated by 0.22 mm laser spot at 300 mm/s (**3**), 200 mm/s (**4**), 100 mm/s (**5**), and 50 mm/s (**6**).

As seen from Table 2, after heating of the HA pellet into an oven at a temperature of 900–1200 °C, the cooled sample has lattice parameters corresponding to a stoichiometric hydroxyapatite. Intensive growth of the HA crystallites takes place up to a temperature of 1200 °C. At a temperature of 1300 °C, a decrease in the crystallite size is observed, which is accompanied by a decrease in the lattice parameter *a* and an increase in the lattice parameter *c* (Table 2). These changes are probably caused by the presence of OH-group vacancies in the HA structure with the formation of the OHA structure according to Reaction (3), which is confirmed by a decrease in the intensity of the stretching and libration vibrations of the OH-group in the FTIR spectrum (Figure 4) [21]. At a temperature of 1400 °C, a further decrease in the lattice parameter *a* and an increase in the lattice parameter *c* occurs; however, at this temperature, the HA crystallite size decreases together with the α-Ca_3_(PO_4_)_2_ phase appearance (Table 2). Apparently, the HA decomposition takes place in the surface layer of the HA particles, where the release of hydroxyl groups is more intense. At a temperature of 1500 °C, the content of the HA phase decreases to 28 wt% and the crystallite size becomes even smaller. These experiments have shown that when the compacted HA powder is heated in a high-temperature furnace, the congruent melting does not occur.

### 3.2. Laser Treatment

#### 3.2.1. Effect of Scanning Speed

**Figure 5:**
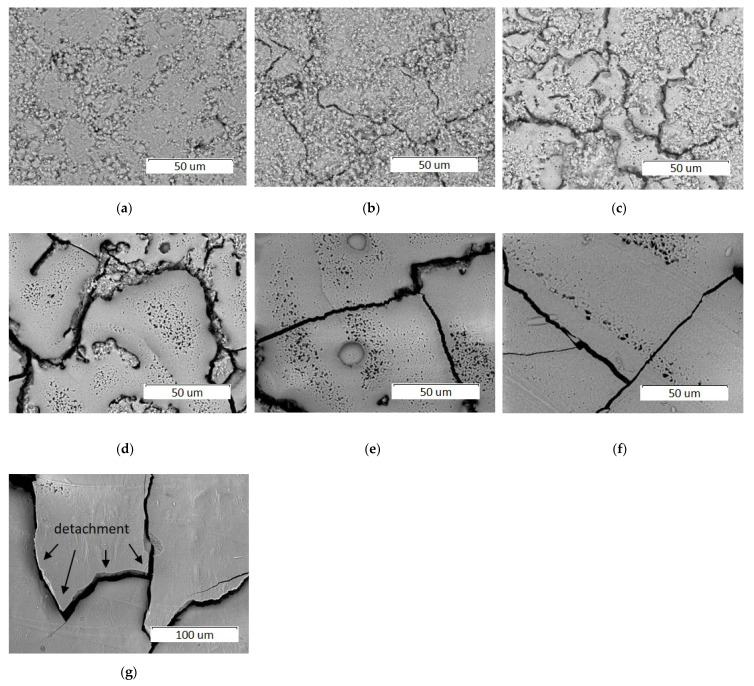
SEM images of pellet surface before (**a**) and after treatment by a 0.25 mm laser spot at a power of 4 W and at different scanning speeds, mm/s: (**b**) 600; (**c**) 500; (**d**) 300; (**e**) 200; (**f**) 100; and (**g**) 50.

Figure 5 shows an image of the surface of pellets treated with laser irradiation at a power of 4 W, at a spot diameter of 0.25 mm, and at a raster pitch of 0.05 mm using different scanning speeds. As seen, a change in the scanning speed, provided that all other parameters remain constant, results in a significant change in the morphology of the sample surface. At a scanning speed of 600 mm/s, the selective laser sintering of HA particles is accompanied with sample shrinkage and cracks formation (Figure 5b). Small droplets of molten material appear at scanning speed of 500 mm/s (Figure 5c). As seen from Table 3, a decrease in the scanning speed leads to an increase in the exposure (i.e., to an increase in the energy transmitted by the laser irradiation to the surface of the sample), and therefore to an increase in the temperature of the particles of the treated material. Taking into account that HA melts at 1570 °C [21], one can assume that the temperature of pellet surface under these conditions reaches this value. A further decrease in scanning speed leads to an increase in the volume of molten material. The maximum coverage of the pellet surface with the recrystallized layer so formed is observed at 100 mm/s (Figure 5f). The microcracks seen in the SEM images are the result of thermal compression of the molten layer that occurs during the cooling process. At a processing speed of 50 mm/s, detachment of the cooled coating occurs.

**Figure 6:**
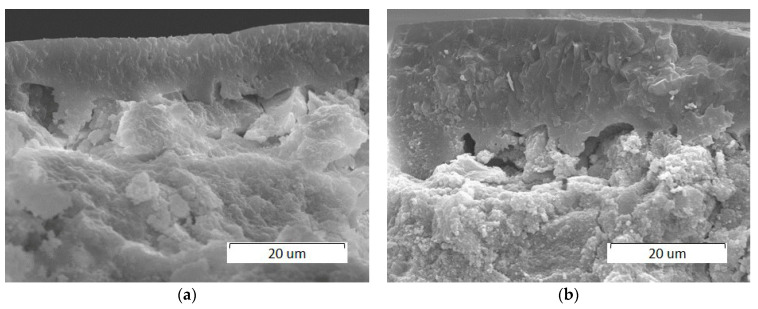
SEM images of cross sections of HA pellets treated by a 0.25 mm laser spot at a power of 4 W and at different scanning speeds: (**a**) 200 mm/s; (**b**) 100 mm/s.

A decrease in the scanning speed results in an increase in the thickness of the molten layer which is approximately 10 µm and 20 µm at 200 and 100 mm/s, respectively (Figure 6). The resulting coating has a dense structure.

**Figure 7:**
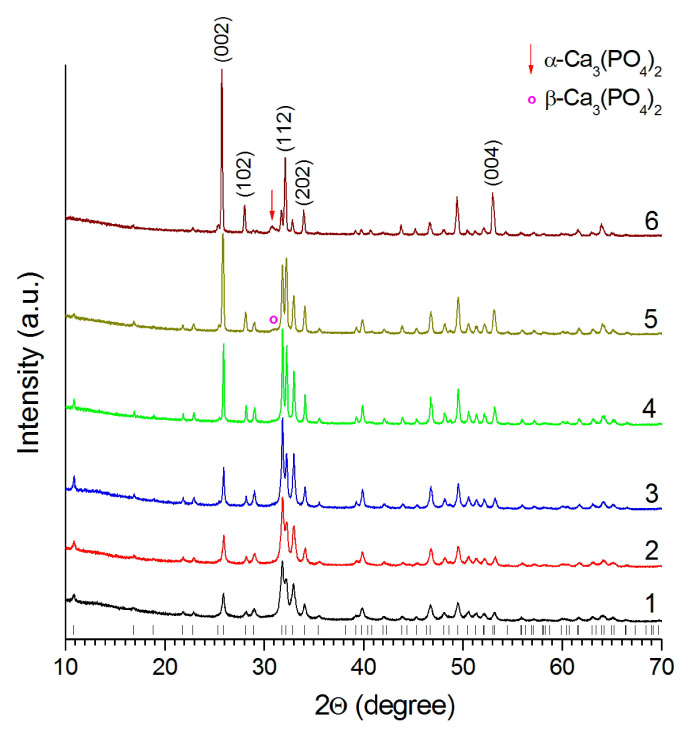
XRD patterns of pellets before (**1**) and after treatment by a 0.25 mm laser at 4 W and different speeds, mm/s: (**2**) 600; (**3**) 500; (**4**) 300; (**5**) 200; and (**6**) 100. Vertical markers correspond to the position of the HA reflections (card PDF 000-09-0432).

The XRD data show that an increase in the amount of melted material results in a significant increase in the intensity of the (002) reflection in the XRD pattern obtained from the laser-treated surface (Figure 7). Obviously, this is due to the formation of a recrystallized layer predominantly oriented in the [00*l*] crystallographic direction. Therefore, the growth of HA crystals occurs along the *c* axis. In addition, it can be seen that at scanning speeds of 200 and 100 mm/s, a barely noticeable reflection of the β-Ca_3_(PO_4_)_2_ and a distinct reflection of the α-Ca_3_(PO_4_)_2_ respectively appear in the XRD patterns, indicating the partial decomposition of HA under these treatment conditions. 

As seen from Table 4, at a scanning speed of 600 mm/s, when HA sintering occurs without melting, the cooled sample is a stoichiometric hydroxyapatite with the corresponding lattice parameters. Consequently, the HA dehydroxylation occurring during laser beam heating is accompanied by rehydroxylation during cooling. At a scanning speed of 400–500 mm/s, when the melting process is just beginning, the recrystallized sample is also a stoichiometric HA with the corresponding lattice parameters. However, at a scanning speed of 300 mm/s, both more intense melting of HA and a decrease in the lattice parameter *a* occur. The FTIR spectra of the resultant coating have low intensity absorption bands of the hydroxyl group (Figure 4), suggesting the recrystallized product is OHA. It can be assumed that rehydroxylation in a thicker recrystallized layer is difficult. Similar to heating in a high-temperature furnace (see Section 3.1), in the case of heating by laser radiation, the as-obtained OHA does not contain impurity phases. A further decrease in the rate of surface treatment (200 mm/s and less) results in the loss of both the hydroxyl groups and the oxide ions located in hexagonal channels and the incongruent melting of OHA takes place.

**Figure 8:**
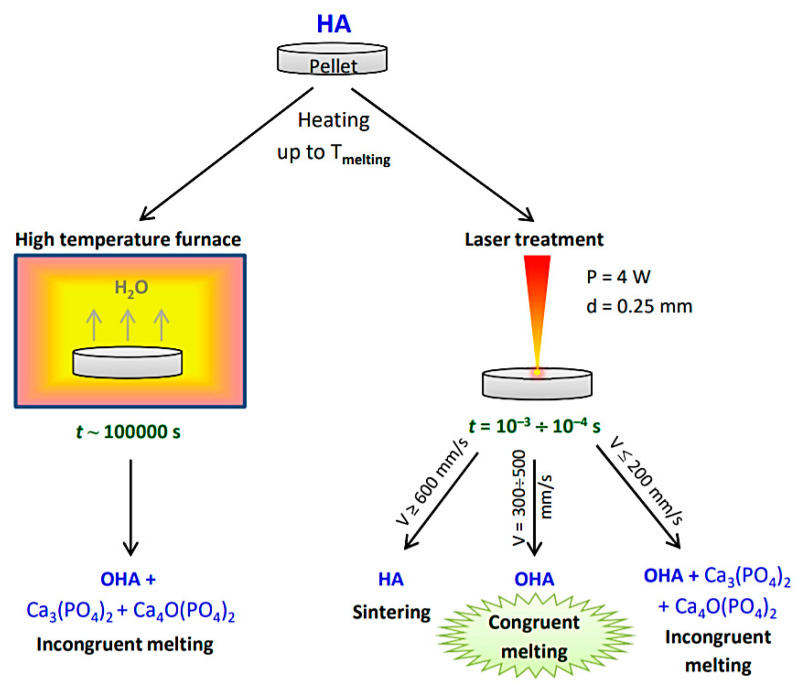
Comparison of options for high-temperature treatment of the HA pellets. P, laser power; d, laser spot diameter; V, scanning speed; *t,* duration of treatment; HA, hydroxyapatite; OHA, oxyhydroxyapatite.

As shown above, the congruent melting of HA by heating in a high-temperature furnace is impossible due to its partial decomposition (at heating rates, as a rule, of no more than 10 °C/min). However, as the current study shows, this can be done by using laser processing with a rapidly moving focused laser beam, characterized by a high heating and cooling rates. The time required for the hydroxyl group to diffuse in the HA nanocrystals is tenths of a second [23]. The heating time of a surface element during laser melting by a scanning beam with a diameter of 0.25 mm and a velocity of 300 mm/s is 8 × 10^−4^ s, which is much shorter than the diffusion time for hydroxyl group. In such a short time, only the surface OH groups can leave the apatite lattice, which makes the congruent melting of HA possible at a scanning speed of 500–300 mm/s (Figure 8). At a scanning speed of 200 mm/s, heating time of a melted zone is 1 × 10^−3^ s, which is also less than it is required for the hydroxyl groups diffusion. Nevertheless, in this case, the partial decomposition of OHA takes place, which is due to the higher exposure value (Table 3) that gives higher point temperature at a scanning speed of 200 mm/s. Since, at low scanning speeds, both the concentration of the decomposition products and the size of apatite crystallites increase as the scanning speed decreases (Table 4), it can be assumed that the decomposition products are formed at the melt crystallization stage.

#### 3.2.2. Effect of Laser Spot Diameter

**Figure 9:**
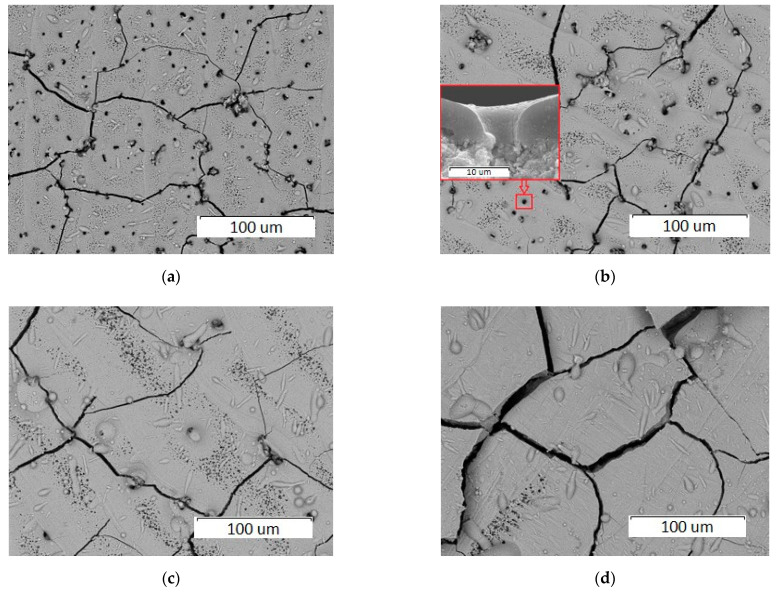
SEM images of pellet surface treated by a 0.2 mm laser spot at 4 W and at different scanning speeds, mm/s: (**a**) 700; (**b**) 500; (**c**) 300; and (**d**) 100. The inset shows a cross section of the specified area.

**Figure 10:**
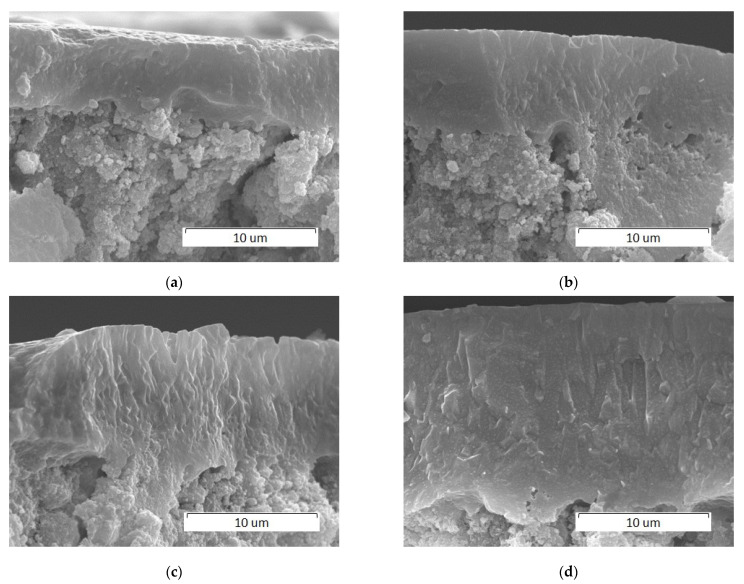
SEM images of cross sections of pellets treated by a 0.2 mm laser spot at 4 W and different scanning speeds, mm/s: (**a**) 700; (**b**) 500; (**c**) 300; and (**d**) 200.

As the laser spot diameter decreases to 0.2 mm, HA melting occurs at a much higher scanning speed, i.e., at 700 mm/s (Figure 9a). Under these conditions, in addition to the cracks formed during the cooling of the melt, a large number of pores with an average diameter of 5 μm are present on the surface of the recrystallized layer. These cavities are assumed to originate from the release of heated air and water vapor (crystallization water, see Reaction 1) from the lower, unmelted layers. This assumption is confirmed by the cavity shape shown in the inset in Figure 9b. As the surface treatment speed decreases, the number of cavities decreases and at a scanning speed of 100 mm/s, cavities are not registered (Figure 9d). However, in this case, a partial detachment of coating is observed. A decrease in the scanning speed results in an increase in the thickness of the recrystallized layer from 7 µm to 15 µm approximately (Figure 10).

The XRD data (Table 4) show that, at a laser spot diameter of 0.2 mm and at laser scanning speeds ranging from 500 to 700 mm/s, the congruent melting of OHA occurs. If the laser spot moves slower, the cooling process slows down and that is evidenced by a slight increase in the crystallite size with a decrease in the scanning speed.

A comparison of the estimated exposure values and processes occurring on the sample surface (sintering, melting or decomposition) indicates (Table 3) that the melting process for 0.2 mm laser spot starts at significantly higher scanning speeds. An increase in the laser irradiation density and, consequently, more intense heating of the sample surface makes it possible to achieve melting at high values of the scanning speed. Accordingly, the value of the threshold exposure for the HA melting process also decreases. A decrease in the laser spot size by 1.25 times makes it possible to increase the scanning speed by 2.3 times. A further decrease in the laser spot size and, consequently, an increase in the scanning speed for selective laser melting of HA were impossible in our experiments due to the limitations of the optical part of the scanning unit.

#### 3.2.3. Effect of Laser Power

As the laser power for both spot sizes increases, incongruent melting with partial destruction of the OHA and the formation of impurity phases occurs (Table 4). Thus, a laser spot size of 0.25 mm and a speed of 600 mm/s allow a sintering process to occur at a power of 4 W, whereas the further increase of power immediately leads to incongruent melting avoiding the congruent melting.

**Figure 11:**
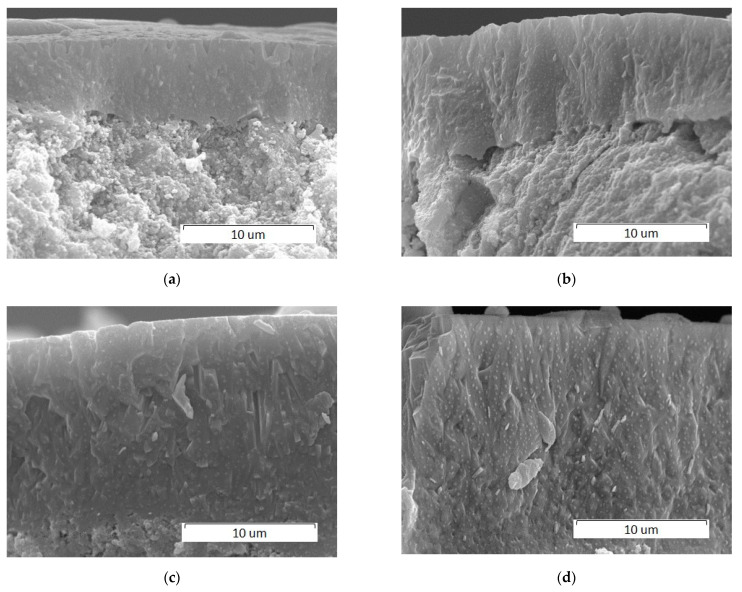
SEM images of cross sections of pellets treated by a 0.2 mm laser spot at a 600 mm/s scanning speed and at different laser powers: (**a**) 4 W; (**b**) 6 W; (**c**) 10 W; and (**d**) 14 W.

The composition of the impurity phases formed at a laser power of more than 4 W for both laser spot sizes is different. At a scanning speed of 600 mm/s and at a laser spot size of 0.25 mm, the decomposition product is the β-Сa_3_(PO_4_)_2_ phase forming in a temperature range of 800–1125 °C [24]. At a laser spot size of 0.2 mm, in addition to the above-mentioned phase, high-temperature phases α-Сa_3_(PO_4_)_2_ and Сa_4_O(PO_4_)_2_ are observed at temperatures above 1125 and 1300 °C, respectively [24]. Consequently, the mode of laser treatment with a spot size of 0.25 mm can be considered to be “softer” as compared to that with a spot size of 0.2 mm. An increase in the laser radiation power leads to an increase in the content of impurity phases. As seen from Figure 11, the thickness of the recrystallized layer increases as the laser power increases. Thus, at a power of 14 W, a layer with a thickness of 20 µm is detected.

It should be noted that, like HA, all of the above impurity phases formed during the HA decomposition under laser irradiation are biocompatible with living tissue and have a higher bioresorption potential than HA [24]. Thus, by varying the laser power, it is possible to change the composition of the printed 3D object and thereby its solubility. The findings of this study offer opportunities to use 3D printing technology for fabrication of implants with a gradient composition, containing layers with different resorption ability.

## 4. Conclusions 

In this study, the behavior of mechanochemically synthesized hydroxyapatite powder during heat treatment in a high-temperature furnace and with laser irradiation has been investigated. The optimal conditions resulting in the formation of a dense monolayer of the recrystallized HA by laser treatment have been determined.

When heating the mechanochemically synthesized hydroxyapatite in a furnace, congruent melting of the material was shown not to occur due to the formation of impurity phases α-Ca_3_(PO_4_)_2_ and Ca_4_O(PO_4_)_2_ at temperatures below the hydroxyapatite melting point.

On the contrary, fast heating of the hydroxyapatite by laser irradiation at a wavelength of 10.6 μm and at a laser spot size of less than 0.3 mm, due to high speed of this process, the congruent melting occurs. It was found that by varying the speed of treatment of the pellet surface at a laser spot size of 0.25 mm and at a laser power of 4 W, different aggregate states of the substance can be reached. At a scanning speed of 600 mm/s, hydroxyapatite is in a solid state and its sintering is accompanied by the growth of crystallites, similar to that when heating in a high-temperature furnace. Congruent melting occurs in the range of scanning speeds of 300–500 mm/s. Under these conditions, a dense recrystallized layer of oxyhydroxyapatite is formed. At speeds of less than 300 mm/s, incongruent melting is observed.

A decrease in the laser spot size significantly shifts the melting threshold of hydroxyapatite towards higher scanning speeds, while maintaining the congruent melting range. This fact is very important for the development of 3D printing technology. It was shown that by treating a layer of the hydroxyapatite powder with a density of 1.9 g/cm^3^, a dense oxyhydroxyapatite layer with an average thickness of 7 μm can be obtained. The number of pores with an average diameter of 5 µm that passing through the recrystallized apatite layer can be controlled by changing the surface treatment speed.

An increase in the laser irradiation power was found to lead to an increase in the thickness of the recrystallized apatite layer. However, in this case, incongruent melting occurs. The concentration of impurity phases increases as the laser power increases. Due to the enhanced bioresorption ability of the resulting impurity phases, the rate of dissolution of the layer so formed can be controlled by varying the laser power. The latter allows the printing of biodegradable implants with a gradient bioresorption variable over the volume of a three-dimensional product.

In summary, we can conclude that the technology of selective laser melting of hydroxyapatite has a great potential for 3D printing of medical biodegradable implants with complex shape and porous gradient structure at sufficiently fast printing speeds. To print an oxyhydroxyapatite object with a layer thickness of 7 μm, the highest possible printing speed of 700 mm/s and the use of a laser spot diameter of 0.2 mm and a power of 4 W are recommended to use. In order to obtain layers with high bioresorption ability, the laser power should be increased up to 14 W. In this case, the thickness of the recrystallized layer can reach 20 µm.

## Figures and Tables

**Table 1:** Parameters of laser treatment of HA pellet surface.

Parameter	Laser Power (W)	Scanning Speed (mm/s)	Scan-Line Pitch (mm)	Laser Spot Size (mm)
Values	4–14	50–700	0.05	0.2, 0.25

**Table 2:** Phase composition and structural parameters of the HA phase of the pellet sintered into the high-temperature furnace at different temperatures and at a humidity of 10%.

Temperature (^o^C)	Content (wt%)			Structural Parameters of the HA Phase		
	α- Ca_3_(PO_4_)_2_	Сa_4_O(PO_4_)_2_	HA	*a* (Å)	*c* (Å)	Crystallite Size (nm)
–	–	–	100	9.4336(12)	6.8932(9)	29.0(4)
900	–	–	100	9.4248(3)	6.8818(2)	116(2)
1000	–	–	100	9.4247(1)	6.8812(1)	245(5)
1200	–	–	100	9.4220(1)	6.8832(1)	345(5)
1300	–	–	100	9.4173(2)	6.8880(2)	256(8)
1400	10	–	90	9.4123(4)	6.8902(4)	138(4)
1500	42	30	28	9.4153(8)	6.8916(10)	116(10)

**Table 3:** Exposure values (J/cm^2^) for different modes of pellet surface treatment at a laser power of 4 W.

Scanning Speed (mm/s)	Diameter of Laser Spot (mm)			
	0.25	Process	0.2	Process
700	11.4	S	11.4	M
600	13.3	S	13.3	M
500	16.0	M	16.0	M
400	20.0	M	20.0	D
300	26.7	M	26.7	D
200	40.0	D	40.0	D

Note: S, sintering; M, melting; D, melting with partial decomposition.

**Table 4:** Phase composition and lattice parameters of the HA phase treated with laser spots of 0.2 and 0.25 mm in size at different scanning speeds and laser power values.

Process	Power (W)/ Speed (mm/s)	Content (wt%)				Structural Parameters of the HA Phase			
		β-TCP	α-TCP	TTCP	HA	*a* (Å)	*c* (Å)	Crystallite Size (nm)	Preferred Orientation
**0.25 mm laser spot**									
S	4/600	–	–	–	100	9.4253(10)	6.8875(8)	33.0(4)	–
M	4/500	–	–	–	100	9.4224(8)	6.8858(6)	47.0(4)	–
M	4/400	–	–	–	100	9.4225(6)	6.8861(6)	57.6(8)	–
M	4/300	–	–	–	100	9.4162(6)	6.8869(4)	90(2)	0.87
D	4/200	3	–	–	97	9.4088(7)	6.8884(5)	80(2)	0.74
D	4/100	7	10	–	83	9.4105(10)	6.8949(6)	117(5)	0.57
D	4/50	–	25	13	62	9.3977(12)	6.9034(8)	120(6)	0.65
D	6/600	2	–	–	98	9.4106(12)	6.8895(8)	64(1)	0.8
D	10/600	6	–	–	94	9.4134(8)	6.8911(5)	115(4)	0.6
D	14/600	9	–	–	91	9.4099(9)	6.8919(5)	136(6)	0.51
**0.2 mm laser spot**									
M	4/700	–	–	–	100	9.4162(8)	6.8879(6)	67(2)	0.76
M	4/600	–	–	–	100	9.4144(8)	6.8886(6)	83(2)	0.74
M	4/500	–	–	–	100	9.4124(8)	6.8895(6)	103(4)	0.65
D	4/400	4	–	–	96	9.4063(8)	6.8868(6)	109(4)	0.58
D	4/300	6	–	–	94	9.4074(10)	6.8925(6)	111(4)	0.51
D	4/200	3	8	–	89	9.4021(10)	6.8948(4)	128(4)	0.32
D	4/100	0	12	7	81	9.4009(9)	6.9017(7)	89(3)	0.61
D	6/600	4	–	–	96	9.4057(12)	6.8882(7)	71(2)	0.56
D	10/600	5	5	6	84	9.4071(9)	6.8965(5)	151(6)	0.48
D	14/600	4	7	16	73	9.4064(18)	6.9014(8)	67(3)	0.48

Notes: S, sintering; M, melting; D, melting with partial decomposition; TCP, Ca_3_(PO_4_)_2_; TTCP, Сa_4_O(PO_4_)_2._

## Data Availability

The raw/processed data required to reproduce these results are included in the Materials and Methods section.
